# In silico comparison of the dosimetric impacts of a greater omentum spacer for abdominal and pelvic tumors in carbon-ion, proton and photon radiotherapy

**DOI:** 10.1186/s13014-019-1411-0

**Published:** 2019-11-21

**Authors:** Masayoshi Yamada, Hiraku Sato, Yoshiro Ieko, Yuya Miyasaka, Takayuki Kanai, Natsuko Yano, Takashi Ono, Hiroko Akamatsu, Mayumi Harada, Mayumi Ichikawa, Yasushi Teranishi, Yasuhiro Kikuchi, Kenji Nemoto

**Affiliations:** 1 Department of Radiation Oncology, Yamagata University Faculty of Medicine, 2-2-2, Iida-Nishi, Yamagata, Japan; 2 Department of Heavy Particle Medical Science, Yamagata University Faculty of Medicine, 2-2-2, Iida-Nishi, Yamagata, Japan; 3 Department of Radiation Oncology, Southern Tohoku Proton Therapy Center, 7-172, Yatsuyamada, Koriyama, Fukushima, Japan; 4 Department of General Surgery, Southern Tohoku Proton Therapy Center, 7-172, Yatsuyamada, Koriyama, Fukushima, Japan

**Keywords:** Spacer, *In silico*, Planning study, Radiotherapy, Particle therapy, Intensity-modulated radiotherapy (IMRT)

## Abstract

**Purpose:**

The purpose of this study was to compare carbon-ion (C-ion), proton and photon radiotherapy (RT) plans with regard to dose reduction of the gastrointestinal (GI) tract by using a greater omentum spacer (GO spacer).

**Methods:**

We retrospectively retrieved data for ten patients who received the GO spacer as surgical spacer placement for abdominal and pelvic tumors. Simulation plans were created on pre-spacer Computed Tomography (CT) and post-spacer CT for C-ion RT, proton RT and photon RT to compare the dose of the GI tract. The plans were normalized so that at least 95% of the planning target volume (PTV) received 70 Gy (relative biological effectiveness equivalent) delivered in 35 fractions. All plans were created with the lowest possible dose to the GI tract under conditions that meet the dose constraints for the PTV and spinal cord (maximum dose < 45 Gy). The part of the GI tract to be evaluated was defined as that most adjacent to the PTV. C-ion RT plans and proton RT plans were calculated by a spot scanning technique, and photon RT plans were calculated employing by fixed-field intensity-modulated radiation therapy.

**Results:**

D2 cc and V10–70 of the GI tract were significantly lower on post-spacer plans than on pre-spacer plans for all three RT modalities. Regarding post-spacer plans, D2 cc of the GI tract was significantly lower on C-ion RT plans and proton RT plans than on photon RT plans (C-ion vs photon *p* = 0.001, proton vs photon *p* = 0.002). However, there was no significant difference between C-ion RT plans and proton RT plans for D2 cc of the GI tract (C-ion vs proton *p* = 0.992). In the photon RT plan for one patient, D2 cc of the GI tract did not meet < 50 Gy.

**Conclusions:**

The GO spacer shows a significant dose reduction effect on the GI tract.

## Background

In radiotherapy (RT) for abdominal and pelvic tumors, the distance between the tumor and gastrointestinal (GI) tract is important for dose prescription. The tolerable dose for the small bowel and large intestine was typically considered to be about 50 Gy [1]. Several previous studies have reported the utility of particle therapy to treat various malignant tumors, including chordoma, rectal cancer (postoperative pelvic recurrence), hepatocellular carcinoma and prostate cancer [2–4]. In these reports, the prescription dose for tumors generally exceeded 50 Gy. In other words, the curative dose for abdominal and pelvic tumors is considered to be higher than the tolerable dose for the GI tract. Therefore, when the tumor is adjacent to the GI tract, it is difficult to deliver a curative dose to the tumor without damaging the adjacent GI tract. The use of a spacer enables separation of the tumor and the GI tract. Thus, by using a spacer, it is possible to safely increase the dose delivered to the tumor and to achieve a curative dose for the tumor.

Methods involving injection of hyaluronic acid or other gels to separate the prostate and rectum were used in previous studies on treatment of prostate cancer [5–12]. Several studies have also been reported that it is possible to safely increase the dose delivered to the tumor by using polytetrafluoroethylene (Gore-tex®) sheets, a tissue expander and the omentum as the spacer [13–17].

Particle therapy has more focused effects than photon RT on target tissues because particle therapy such as carbon-ion (C-ion) RT and proton RT has a Bragg peak. As described above, several studies have shown the utility of particle therapy for treatment of various malignant tumors including chordoma, rectal cancer (postoperative pelvic recurrence), hepatocellular carcinoma and prostate cancer [2–4]. Recently, the use of particle therapy for abdominal and pelvic tumors has been increasing. However, there has been no study in which the dose distributions of C-ion RT, proton RT and photon RT for patients with abdominal and pelvic tumors were compared. This study was an in silico planning comparative study that was carried out to compare C-ion RT, proton RT and photon RT plans regarding dose reduction of the GI tract by the use of a greater omentum spacer (GO spacer).

## Methods

### Study population

**Fig. 1:**
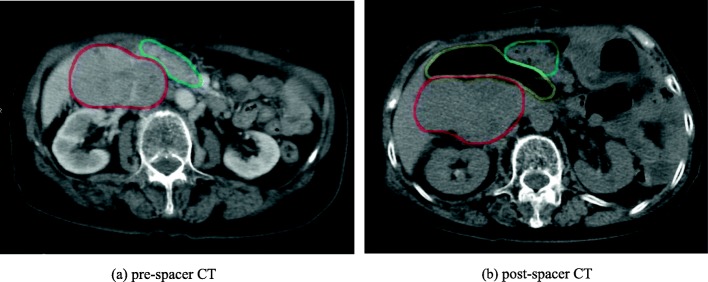
Example of pre-spacer CT (**a**) and post-spacer CT (**b**). The lines show the spacer (yellow), the tumor (red) and duodenum (blue)

We retrospectively retrieved data for ten patients who received surgical spacer placement for abdominal and pelvic tumors in Southern Tohoku Proton Therapy Center from February 2009 through October 2016. In all patients, the tumor was adjacent to the GI tract, and GO spacer was inserted between the tumor and surrounding GI tract by laparotomy. Simulation plans were created on pre-spacer Computed Tomography (CT) and post-spacer CT for all three RT modalities (i.e., C-ion RT, proton RT and photon RT). Post-spacer CT images were taken within 4 weeks after surgery. Pre-spacer CT images were mainly acquired using a 64-row detector CT system (Optima 660, General Electric Medical Systems, Milwaukee, USA) or a 4-row detector positron emission tomography (PET)-CT system (Discovery LS, General Electric Medical Systems, Milwaukee, USA), but some pre-spacer CT images were acquired in other facilities (CT details unknown). Post-spacer CT images were acquired using a 16-row detector CT system (Aquilion LB, Canon Medical Systems, Otawara, Japan). Figure 1 shows an example of pre-spacer CT and post-spacer CT images. All of the patients were positioned supine. A set of 3–5-mm-thick CT images was taken for pre-spacer CT and a set of 2–5-mm-thick CT images was taken for post-spacer CT.

### Target delineation and treatment planning

All contouring and treatment plans were created by one certified radiation oncologist with 6 years of radiation therapy experience and were confirmed by two medical physicists with more than 1 year of C-ion RT experience.

MIM ver. 6.4.6 (MIM Software Inc., Cleveland, OH) was used for contouring. The gross tumor volume (GTV) was delineated as the macroscopic tumor on CT and/or hybrid fluorodeoxyglucose (FDG)-PET. The clinical target volume (CTV) was the same as GTV. The planning target volume (PTV) included the CTV with a 7-mm margin for possible positioning errors. Assuming the usage of respiratory gating for upper abdominal tumors, internal margin for compensating respiratory motion was regarded to be sufficiently small. The GI tract (duodenum, small bowel and colon) and spinal cord were defined as organs at risk (OARs). The GI tract was delineated in the range expanded 10 mm to the cranial and caudal sides from the PTV. Clinical delineations that were performed on pre-spacer CT and post-spacer CT by experienced radiation oncologists were used in this study.

C-ion RT plans were calculated on the VQA treatment planning system (TPS) ver. 3.2 (Hitachi, Ltd., Japan). C-ion RT plans were calculated by a spot scanning technique based on irradiation equipment of Heavy-Ion Medical Accelerator in Chiba (HIMAC) [18]. The single-field uniform-dose (SFUD) optimization technique was applied [19]. Relative biological effectiveness (RBE) was taken into account using the spread-out Bragg peak (SOBP) concept to calculate the dose in Gy (RBE) based on irradiation equipment of HIMAC [20]. The dose was calculated using a pencil beam algorithm.

Proton RT plans were calculated on the VQA TPS. Proton RT plans were also calculated by a spot scanning technique based on irradiation equipment of Nagoya Proton Therapy Center [21]. The SFUD optimization technique was applied. The dose was calculated using a pencil beam algorithm, assuming an RBE of 1.1 for proton [22].

Photon RT plans were calculated on EclipseTM ver.11.0 (Varian Medical Systems, Palo Alto, CA). Photon RT plans were calculated using fixed-field intensity-modulated radiotherapy (IMRT) with 10 MV photons. The dose was calculated with the analytical anisotropic algorithm [23].

Seventy Gy (RBE equivalent) delivered in 35 fractions to the PTV was prescribed in all plans. The plans were normalized so that at least 95% of the PTV received the prescribed dose. The dose fraction number in C-ion RT is small, but in order to compare C-ion RT, proton RT and photon RT plans, the prescribed dose was unified at 2 Gy (RBE) per fraction. The dose constraint for the spinal cord was maximum dose (Dmax) < 45 Gy. All plans were created with the lowest possible dose to the GI tract under conditions that meet the dose constraint for the PTV and spinal cord. We aimed to satisfy the dose constraints of the minimum dose received by the most exposed 2 cc volume of the organ (D2 cc) of the GI tract < 50 Gy if possible. For each plan, we chose the coplanar beam arrangement that minimizes the dose to the GI tract as much as possible. C-ion RT and proton RT plans consisted of one to three beams, and IMRT plans consisted of three to five beams. The optimal number of beams and beam angles were investigated with pre-spacer plans and post-spacer plans so that the D2cc of the GI tube is minimized in each plan. The same number of beams and the same beam angles were used for C-ion RT and proton RT. All plans of the three RT modalities were calculated with the assumption of using a rotating gantry.

### Dose evaluation and statistical analysis

Dose volume histograms (DVHs) were generated for the PTV and all OARs. D98% (minimum dose to ‘hottest’ 98% of the volume), Dmean (mean dose), D50% (median dose), D2% (minimum dose to ‘hottest’ 2% of the volume) and homogeneity index (HI) of the PTV were evaluated on pre-spacer plans and post-spacer CT plans for all three RT modalities. HI was defined by the following equation: HI = (D2%-D98%)/D50%. In this study, the separation distance from the GI tract to the PTV was defined as the shortest distance without overlap by expanding the PTV in three dimensions. In patients with multiple parts of the GI tract adjacent to the PTV, the part of the GI tract with a large overlap volume between the GI tract and the PTV on pre-spacer CT images was selected for evaluation. The relationship between D2 cc of the GI tract and separation distance from the GI tract to the PTV was evaluated on post-spacer plans.

MIM was used for dose evaluation. We tested the differences in mean values of D2 cc of the GI tract, V10, V15, V20, V25, V30, V35, V40, V45, V50, V55, V60, V65 and V70 (Vx was used to mean the volumes at least received x Gy) of the GI tract and HI of the PTV on pre-spacer CT and post-spacer CT images for all three RT modalities. These values were analyzed statistically by Wilcoxon’s signed-rank test with a *p*-value < 0.05 considered statistically significant. Because the presented sample size is very small, we performed a cross validation test using the leave-one-out method on D2 cc and Vx of the GI tract that showed significant differences in Wilcoxon’s signed-rank test. Statistical analysis was performed using SPSS ver.23 (IBM Corp., New York, NY; formerly SPSS Inc., Chicago, IL).

## Results

Patient characteristics are shown in Table 1. Tumor localization in patients 1–5 was around the liver and that in patients 6–10 was in the pelvic region.

**Table 1:** Patient characteristics

Patient	Name of disease	Tumor localization	Pre-spacer PTV volume (cc)	Post-spacer PTV volume (cc)	Separation Distance^a^ (cm)	Part of the GI tract most adjacent to the PTV on post-spacer CT
1	Liver metastasis (ascending colon cancer)	Right lobe of the liver	335.3	223.8	0.24	Transverse colon
2	Hepatocellular carcinoma	Right lobe of the liver	25.1	34.1	0.24	Transverse colon
3	Hepatocellular carcinoma	Right lobe of the liver	723.0	807.2	2.00	Transverse colon
4	Leiomyoma	Inferior vena cava	730.2	729.3	0.72	Duodenum
5	Leiomyoma	Right lobe of the liver	174.6	200.4	1.14	Duodenum
6	Local recurrence (sigmoid colon cancer)	Pelvic floor	37.9	42.6	8.78	Sigmoid colon
7	Bone metastasis (uterine corpus cancer)	Left acetabular & sacrum	443.2	470.8	0.43	Sigmoid colon
8	Local recurrence (rectal cancer)	Pelvic floor	262.4	265.9	1.25	Small bowel
9	Lymph node metastasis (cancer of unknown primary)	Left side of the pelvis	3022.2	3575.7	1.90	Small bowel
10	Chordoma	Sacrum	283.7	259.8	4.04	Small bowel

^a^The separation distance from the GI tract to the PTV was defined as the shortest distance without overlap by expanding the PTV in three dimensions

*Abbreviations*: *PTV* Planning target volume, *GI tract* Gastrointestinal tract

**Fig. 2:**
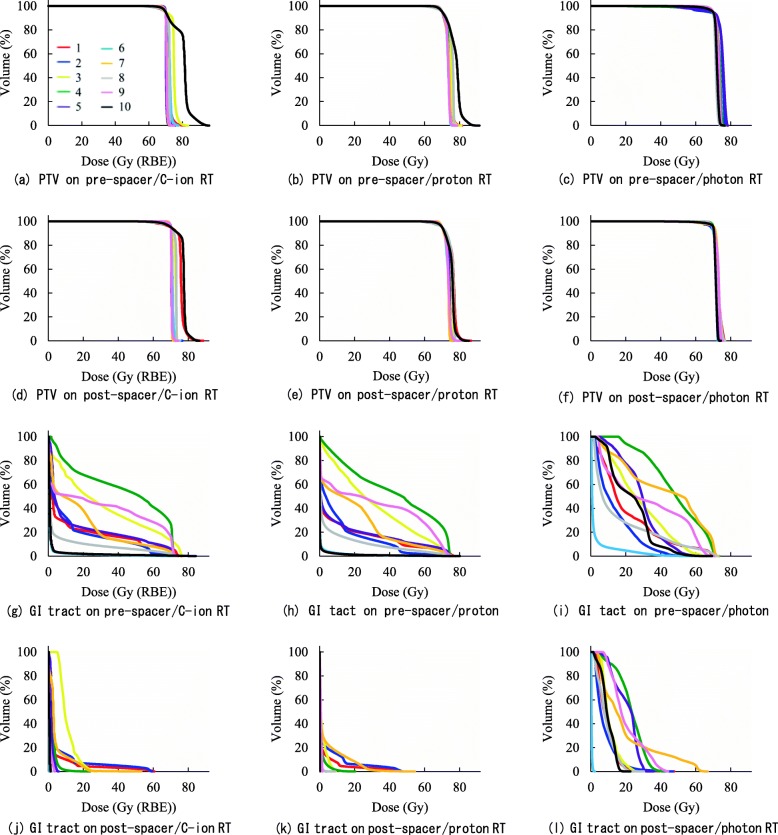
DVHs for the PTV and GI tract on pre-spacer plans and post-spacer plans with color coding in each case: (**a**) PTV on pre-spacer/C-ion RT, (**b**) PTV on pre-spacer/proton RT, (**c**) PTV on pre-spacer/photon RT, (**d**) PTV on post-spacer/C-ion RT, (**e**) PTV on post-spacer/proton RT, (**f**) PTV on post-spacer/photon RT, (**g**) GI tract on pre-spacer/C-ion RT, (**h**) GI tact on pre-spacer/proton RT, (**i**) GI tact on pre-spacer/photon RT, (**j**) GI tract on post-spacer/C-ion RT, (**k**) GI tract on post-spacer/proton RT, and (**l**) GI tract on post-spacer/photon RT

**Fig. 3:**
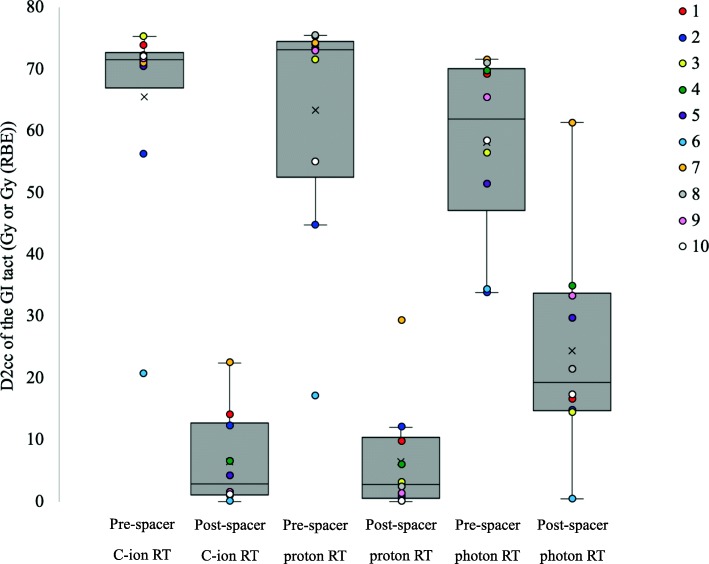
Multiple box plots and dose plots (color-coded) of D2cc of the GI tract. The box plots represent data with boxes ranging from the 25th to the 75th percentile of the observed distribution of values. Horizontal lines represent the median values for D2 cc of the GI tract. X marks represent the mean values for D2 cc of the GI tract. Whiskers span minimum to maximum observed values with algorithm-defined outliers. Dose plots of D2 cc values for all three modalities in pre-spacer plans and post-spacer plans were color-coded in each case and overlaid on the box plots

For each plan, all DVHs for the PTV and spinal cord met constraints respectively. Differences in plan parameter (mean (SD)) for all three RT modalities are shown in Table 2. There was no significant difference in HI of the PTV between pre-spacer plans and post spacer plans for each of three RT modalities (C-ion *p* = 0.344, proton p = 0.344, photon p = 0.344). Regarding pre-spacer plans, there was no significant difference in HI of the PTV between all three RT modalities (C-ion vs proton *p* = 0.754, C-ion vs photon *p* = 1.000, proton vs photon p = 1.000). Regarding post-spacer plans, there was no significant difference in HI of the PTV between all three RT modalities (C-ion vs proton p = 0.344, C-ion vs photon p = 0.754, proton vs photon *p* = 0.109). DVHs for the PTV and GI tract on pre-spacer plans and post-spacer plans for all three RT modalities are shown in Fig. 2. Multiple box plots and dose plots (color-coded) of D2cc of the GI tract on pre-spacer plans and post-spacer plans for all three RT modalities are summarized in Fig. 3.The spacer significantly reduced D2 cc of the GI tract for C-ion RT (mean ± SD: 65.4 ± 5.2 Gy vs 6.4 ± 2.4 Gy, *p* < 0.0001), proton RT (63.3 ± 6.1 Gy vs 6.4 ± 2.9 Gy, p < 0.0001) and photon RT (58.1 ± 4.5 Gy vs 24.4 ± 5.2 Gy, p < 0.0001). Regarding post-spacer plans, D2 cc of the GI tract was significantly lower on C-ion RT plans and proton RT plans than on photon plans (C-ion vs photon *p* = 0.001, proton vs photon *p* = 0.002). However, there was no significant difference between C-ion RT plans and proton RT plans for D2 cc of the GI tract (C-ion vs proton *p* = 0.992).

**Table 2:** Differences in plan parameter (mean (SD)) for all three RT modalities

	Measure	C-ion RT		Proton RT		Photon RT	
		Pre-spacer	Post-spacer	Pre-spacer	Post-spacer	Pre-spacer	Post-spacer
PTV	D98% (Gy)	68.2 (0.44)	67.7 (0.75)	68.4 (0.24)	68.2 (0.36)	64.9 (1.52)	67.6 (0.51)
	Dmean (Gy)	72.5 (0.97)	72.4 (0.79)	74.3 (0.44)	74.2 (0.33)	72.7 (0.32)	72.1 (0.17)
	D50% (Gy)	72.7 (1.07)	72.6 (0.86)	74.9 (0.51)	74.7 (0.39)	73.3 (0.43)	72.4 (0.19)
	D2% (Gy)	75.4 (1.92)	74.7 (1.42)	76.8 (1.01)	76.7 (0.69)	74.9 (0.45)	74.0 (0.38)
	HI	0.096 (0.03)	0.093 (0.03)	0.111 (0.01)	0.113 (0.01)	0.135 (0.01)	0.089 (0.01)
Spinal cord	Dmax (Gy)	4.3 (2.82)	3.5 (2.10)	5.4 (3.77)	4.1 (2.74)	14.8 (6.09)	13.3 (5.31)

*Abbreviations*: *Dmean* Mean dose, *D2%* Minimum dose to ‘hottest’ 2% of the volume;

*D98%* Minimum dose to ‘hottest’ 98% of the volume, *Dmax* Maximum dose

**Fig. 4:**
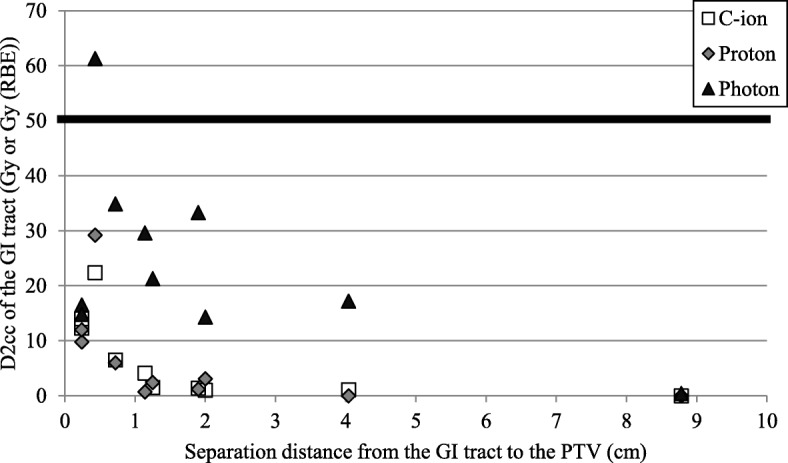
Relationship between D2 cc of the GI tract and separation distance on post-spacer plans

**Fig. 5:**
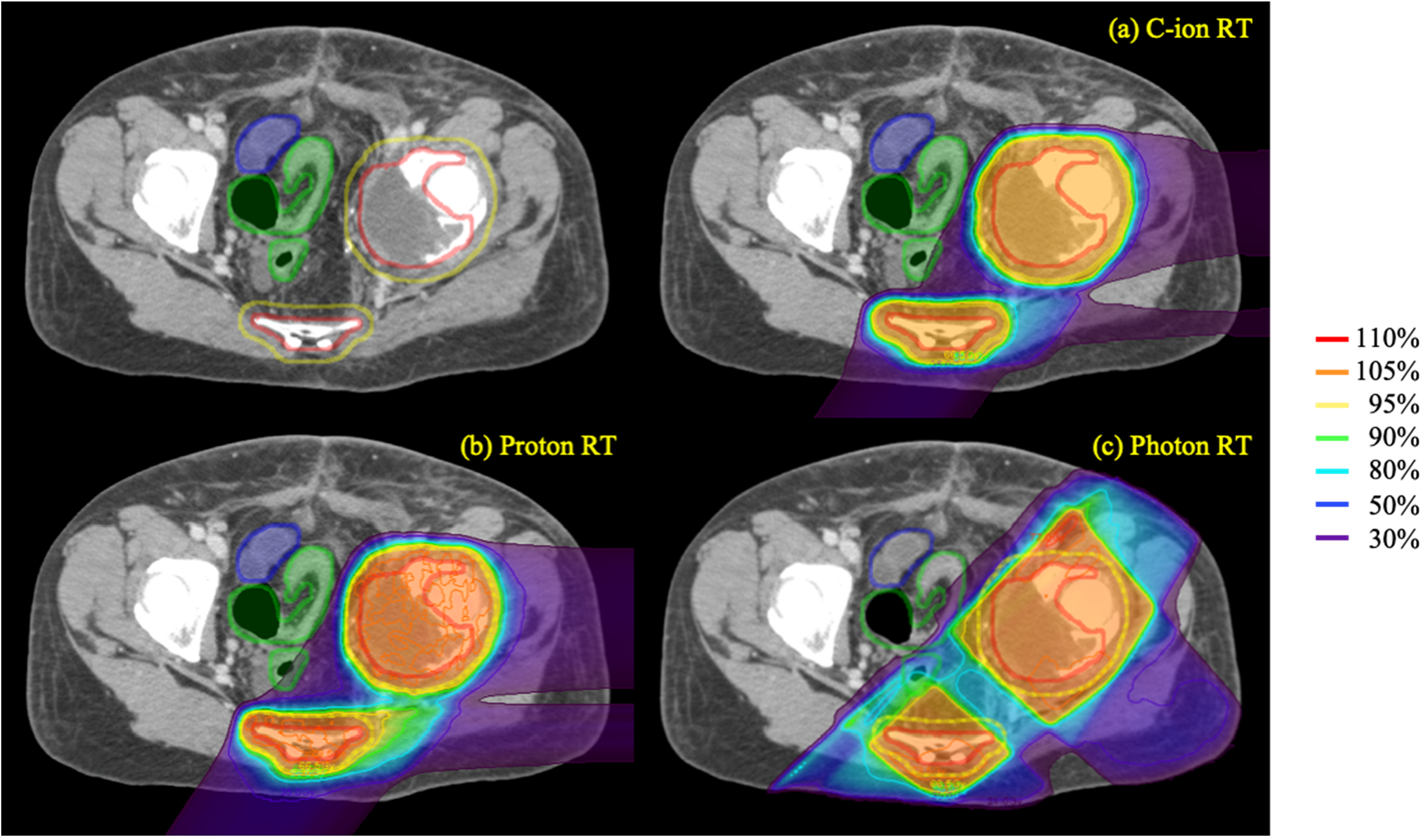
Dose distributions on post-spacer plans of all three RT modalities for patient 7: (**a**) C-ion RT, (**b**) proton RT, and (**c**) photon RT. The lines show the spacer (orange), tumor (red), PTV (yellow), bowel (blue) and colon (green)

The relationship between D2 cc of the GI tract and separation distance from the GI tract to the PTV on post-spacer plans is shown in Fig. 4. As described above, the separation distance from the GI tract to the PTV was defined as the shortest distance without overlap by expanding the PTV in three dimensions. In the photon RT plan for one patient (patient 7), D2 cc of the GI tract did not meet the constraint. Because the separation distance from the GI tract to the PTV on post-spacer plans of patient 7 was not the shortest among all patients, the spacer thickness required to effectively reduce the dose of the GI tract could not be examined. The GTV of that patient was left acetabular and sacral bone metastasis of uterine body cancer. In that patient, the part of the GI tract that was most adjacent to the PTV on post-spacer CT was the sigmoid colon and the separation distance from the PTV to the GI tract was 0.43 cm. In that patient, D2 cc values for the GI tract of C-ion RT, proton RT and photon RT were 22.4, 29.2 and 61.3 Gy, respectively. Figure 5 shows dose distributions on post-spacer plans of all three RT modalities for that patient.

**Fig. 6:**
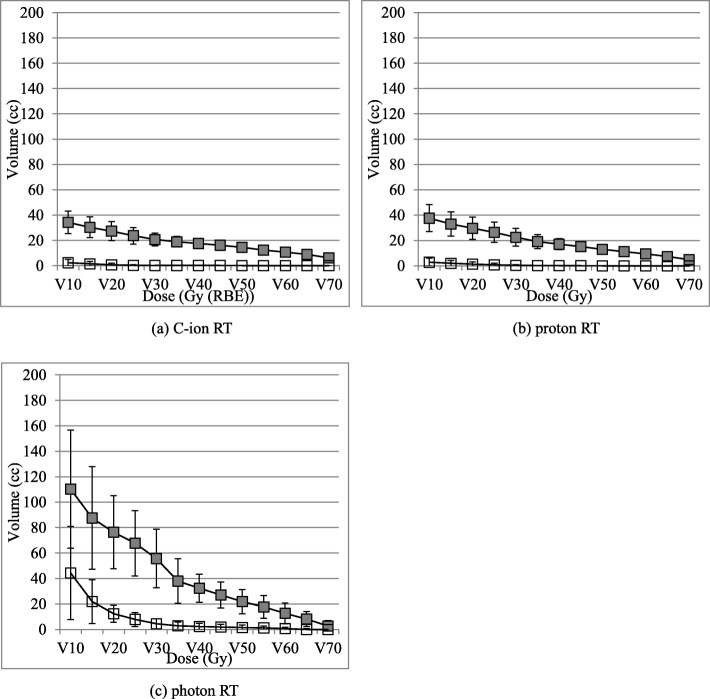
V10–70 (cc) means ± SD to the GI tract on pre-spacer and post-spacer plans: (**a**) C-ion RT, (**b**) proton RT and (**c**) photon RT: The error bars indicated SD of the population

V10–70 means ± SD of the GI tract in all patients are summarized in Fig. 6. The spacer significantly reduced V10-V70 of the GI tract for all treatment modalities (all *p*-values < 0.05). Reduction of V10 and V15 of the GI tract by the spacer was more effectively achieved by C-ion RT and proton RT than by photon RT. However, there was no significant difference between C-ion RT and proton RT in reduction of V20-V70 of the GI tract by the spacer.

Because the presented sample size is very small (only 10 cases), we performed a cross validation test using the leave-one-out method on D2 cc and Vx of the GI tract that showed significant differences in Wilcoxon’s signed-rank test and confirmed the statistical difference (all p-values < 0.05).

## Discussion

In previous studies comparing the dose distribution of IMRT techniques for HCC, the conformity index values showed no significant difference between helical-IMRT and static-IMRT (e.g., fixed-field IMRT), but the homogeneity index values significantly improved with helical-IMRT compared with static-IMRT [24, 25]. It has also been reported that the mean dose and low dose region of the normal liver in helical-IMRT were higher than static-IMRT [26]. The purpose of this study was to reduce the dose in the adjacent gastrointestinal tract. Therefore, as photon treatment plans, we chose fixed-field IMRT which was considered to have lower dose to the surroundings, instead of helical-IMRT which was considered to have high homogeneity index values for PTV.

Several studies in which the rectal dose with a spacer for prostate cancer was examined showed that the spacer significantly reduced Dmax, Dmean, and V70 of the rectum [5, 8, 12]. However, there are only case reports of studies showing dose reduction of the GI tract by a spacer for abdominal and pelvic tumors other than prostate cancer [13, 27–29]. Therefore, this study is the first study in which the dosimetric effect of a spacer on the GI tract was examined in detail.

We showed that the spacer significantly reduced D2 cc of the GI tract for all three RT modalities. Reduction of D2 cc of the GI tract was more effectively achieved by C-ion RT and proton RT than by photon RT. In the photon RT plan for one patient, D2 cc of the GI tract did not meet the constraint. In that case, it is thought that the PTV was a complicated shape with a large area in contact with the GI tract and that photon RT without a Bragg peak was unable to reduce the dose of the GI tract.

It is noteworthy that there was no significant difference between C-ion RT and proton RT in reduction of D2 cc of the GI tract by the GO spacer. In general, the distal dose to C-ion RT is considered to be higher than that to proton RT due to the fragmentation tail of C-ions [20, 30, 31]. Also, the lateral dose to C-ion RT has been reported to be lower than that to proton RT [32–34]. In this study, a comparison of C-ion RT plans and proton RT plans with regard to D2 cc of the GI tract on post-spacer plans showed that there were cases where C-ion RT was superior and there were cases where proton RT was superior. This is thought to depend on the positional relationship of the GI tract and the tumor with respect to the beam angle. However, it is not clear as the number of cases in this study was small.

In this study, the tumor location (upper abdomen vs pelvis) and the type of adjacent GI tract (duodenum/small intestine vs colon) were examined for an impact on the results and conclusions. However, no findings that could affect the results and conclusions were obtained. This point should be further studied in the future work.

By a search using the keywords “spacer, radiation” in PubMed, we found various reports on the use of hydrogel for prostate cancer and the use of Gore-tex® sheets and a tissue expander for abdominal and other pelvic tumors. Hydrogel is gradually absorbed, but Gore-Tex® and a tissue expander are not absorbed and the latter must be removed after radiation therapy. Migration of artificial materials into the small bowel was described in case reports [35, 36]. Ogino et al. reported that migration of the spacer and small bowel obstruction occurred in a patient in whom Gore-tex® was used as the spacer [35]. Thus, complications can occur due to the foreign nature of the spacer. Multiple cases using the omentum as the spacer for proton therapy have been reported [17]. In this study, the greater omentum was inserted as the spacer. However, there has been no discussion about its advantages and disadvantages.

One advantage of a GO spacer is that it might reduce the risk of complications (e.g., migration and infection) because the greater omentum is an in vivo material. One disadvantage of a GO spacer is that it might be difficult to use a GO spacer for cases other than abdomen and pelvic tumors, cases with an operative history of the abdomen and pelvic area, and cases of a large tumor that was difficult to cover all around with a greater omentum spacer. In other words, cases in which a GO spacer can be used are limited. For such cases, an artificial material such as Gore-Tex® or a tissue expander must be used as the spacer, but the risk of complications as described above must be considered.

Recently, research on a bioabsorbable spacer using polyglycolic acid (PGA) has advanced [37]. If a bioabsorbable spacer is put to practical use, there is a possibility of solving the problems that can occur when the greater omentum and artificial materials are used as spacers.

There are some limitations of this study. First, there were patients in whom the tumor shrank after the spacer surgery. The cause was excision of a part of the tumor during the spacer surgery or the use of an anticancer drug. Second, CT slice thickness was not uniform and was not small. We think that the slice thickness of CT should be uniform and small. However, it was not possible to use a uniform thin slice CT because this study was conceived after the patients had been treated. Third, 4D-CT was typically considered for RT with upper abdominal tumors. However, this study was conceived after the patients had been treated. Because RT for patients with upper abdominal tumors was assumed to use respiratory gating, the treatment plans were created assuming that the internal margin was included in the PTV margin. Fourth, we did not examine robust planning in particle therapy. In this study, isotropic margins were used for all treatment modalities to simplify comparison of dose distributions for C-ion RT, proton RT and photon RT. Also, the beam angles to hit the tumor after passing through the spacer were not used because the spacer was adjacent to the GI tract. Therefore, we decided not to change the margin with or without spacer. Fifth, we did not impose dose constraints on the liver and kidneys. The aim of this study was to determine the effectiveness of the spacer for the GI tract. When we imposed dose constraints on the liver and kidneys as described in Emami B et al. [1], all five cases for C-ion and proton RT plans met those, however, two cases (cases 3 and 4) of five cases of upper abdominal tumors for photon RT plans (both pre- and post-spacer plans) did not meet dose constraint of the liver. Cases 3 and 4 with large tumor for photon RT plans did not meet dose constraint of the liver, therefore cases with large tumor for photon plans may not meet the dose constraint of the liver with or without the spacer.

## Conclusions

The GO spacer shows a significant dose reduction effect on the GI tract for C-ion RT, proton RT and photon RT.

## Data Availability

The datasets analyzed during the current study are available from the corresponding author on reasonable request.

## Abbreviations

C-ion: Carbon-ion
CT: Computed Tomography
CTV: Clinical target volume
D2 cc: The most exposed 2 cc volume of the organ
D2%: Minimum dose to ‘hottest’ 2% of the volume
D50%: Median dose
D98%: Minimum dose to ‘hottest’ 98% of the volume
Dmax: Maximum dose
Dmean: Mean dose
DVHs: Dose volume histograms
EQD2: Equivalent dose in 2 Gy fractions
FDG-PET: Hybrid fluorodeoxyglucose positron emission tomography
GI: Gastrointestinal
GO spacer: The greater omentum spacer
GTV: Gross tumor volume
HI: Homogeneity index
HIMAC: Heavy-Ion Medical Accelerator in Chiba
IMRT: Intensity-modulated radiation therapy
OARs: Organs at risk
PTV: Planning target volume
RBE: Relative biological effectiveness
RT: Radiotherapy
SFUD: Single-field uniform-dose
SOBP: Spread-out Bragg peak
TPS: Treatment planning system
Vx: The volumes at least received x Gy
